# Immunotoxin-Mediated Tract Targeting in the Primate Brain: Selective Elimination of the Cortico-Subthalamic “Hyperdirect” Pathway

**DOI:** 10.1371/journal.pone.0039149

**Published:** 2012-06-25

**Authors:** Ken-ichi Inoue, Daisuke Koketsu, Shigeki Kato, Kazuto Kobayashi, Atsushi Nambu, Masahiko Takada

**Affiliations:** 1 Systems Neuroscience Section, Primate Research Institute, Kyoto University, Inuyama, Japan; 2 Division of System Neurophysiology, National Institute for Physiological Sciences and Department of Physiological Sciences, The Graduate University for Advanced Studies, Okazaki, Japan; 3 Department of Molecular Genetics, Institute of Biomedical Sciences, Fukushima Medical University School of Medicine, Fukushima, Japan; Centre national de la recherche scientifique, France

## Abstract

Using a neuron-specific retrograde gene-transfer vector (NeuRet vector), we established immunotoxin (IT)-mediated tract targeting in the primate brain that allows ablation of a neuronal population constituting a particular pathway. Here, we attempted selective removal of the cortico-subthalamic “hyperdirect” pathway. In conjunction with the direct and indirect pathways, the hyperdirect pathway plays a crucial role in motor information processing in the basal ganglia. This pathway links the motor-related areas of the frontal lobe directly to the subthalamic nucleus (STN) without relay at the striatum. After electrical stimulation in the motor-related areas such as the supplementary motor area (SMA), triphasic responses consisting of an early excitation, an inhibition, and a late excitation are usually detected in the internal segment of the globus pallidus (GPi). Several lines of pharmacophysiological evidence suggest that the early excitation may be derived from the hyperdirect pathway. In the present study, the NeuRet vector expressing human interleukin-2 receptor α-subunit was injected into the STN of macaque monkeys. Then, IT injections were made into the SMA. In these monkeys, single-neuron activity in the GPi was recorded in response to the SMA stimulation. We found that the early excitation was largely reduced, with neither the inhibition nor the late excitation affected. The spontaneous firing rate and pattern of GPi neurons remained unchanged. This indicates that IT-mediated tract targeting successfully eliminated the hyperdirect pathway selectively from the basal ganglia circuitry without affecting spontaneous activity of STN neurons. The electrophysiological finding was confirmed with anatomical data obtained from retrograde and anterograde neural tracings. The present results define that the cortically-driven early excitation in GPi neurons is mediated by the hyperdirect pathway. The IT-mediated tract targeting technique will provide us with novel strategies for elucidating various neural network functions.

## Introduction

To define the framework of complex and elaborate neural networks, it is essential to systematically understand diverse brain functions acquired on network basis. For elucidating the functional role of a given pathway, it is an effective approach to examine behavioral/physiological deficits due to ablation of a neuronal population that forms the target pathway. Cell targeting mediated by immunotoxin (IT) has been developed in mice as a genetic method for ablating distinct types of neurons from neural networks and applied to identify their specific roles [1]–[3]. Recently, we have found that the use of modified glycoprotein of rabies virus for a pseudotyped lentiviral vector based on human immunodeficiency virus type 1 (HIV-1) can enhance the efficiency of gene transfer through retrograde transport of the vector [4], [5]. This property of the pseudotyped vector is greatly meritorious for gene transfer into cell bodies of neurons that are located remote from the injection site of the vector. For IT-mediated targeting of a particular pathway, the highly-efficient retrograde gene-transfer vector was prepared to express human interleukin-2 receptor α-subunit (IL-2Rα), a receptor molecule for the recombinant IT, in neuronal cell bodies through retrograde transport of the vector. In mice with the vector expressing IL-2Rα injected into the striatum, IT injection into the thalamus indeed succeeded in selective removal of the thalamostriatal pathway [6].

We applied the IT-mediated tract targeting technique to the primate brain, because the use of nonhuman primates as animal models is crucial for exploring higher brain functions. Utilizing the dopaminergic nigrostriatal pathway as a test system, we have recently established the basic methodology with a neuron-specific retrograde gene-transfer vector (NeuRet vector) that we have newly developed with improved neuronal specificity [7]. By injecting the NeuRet vector expressing IL-2Rα and IT respectively into the striatum and the substantia nigra in macaque monkeys, we anatomically verified the validity of the tract targeting technique [8]. In the present study, an attempt was made to eliminate the cortico-subthalamic “hyperdirect” pathway selectively from the basal ganglia circuitry in macaque monkeys (for the hyperdirect pathway, see Refs. 9,10). The subthalamic nucleus (STN) receives input from the cerebral cortex, especially from the motor-related areas of the frontal lobe and, in turn, sends output to the internal segment of the globus pallidus (GPi), a major output station of the basal ganglia [9]–[15]. It has been shown that electrical stimulation in the motor-related areas, including the primary motor cortex (MI) and the supplementary motor area (SMA), induces an early, short-latency excitation in GPi neurons, followed by an inhibition and then a late, long-latency excitation [10], [16], [17]. Based on several pharmacophysiological findings, the early excitation is considered to be derived from the cortico-STN-GPi pathway [10], [16], [17], although no direct evidence has as yet been available. Employing IT-mediated tract targeting, we addressed the issue on the contribution of this hyperdirect pathway to the emergence of the early excitation.

## Materials and Methods

### Viral Vector Production

The details of the NeuRet vector were described in our previous paper [7]. The NeuRet vector is a pseudotype of an HIV-1-based lentiviral vector with fusion glycoprotein C type (FuG-C), in which a short C-terminal segment of the extracellular domain and the transmembrane/cytoplasmic domains of rabies virus glycoprotein are replaced with the corresponding regions of vesicular stomatitis virus glycoprotein. The NeuRet vector enhances the efficiency of retrograde gene transfer into neuronal cells with less efficiency of gene transduction into dividing cells in the brain. The envelope plasmid contained FuG-C cDNA under the control of cytomegalovirus enhancer/chicken β-actin promoter. The transfer plasmids contained the cDNA encoding IL-2Rα fused to enhanced green fluorescent protein (IL-2Rα-GFP) downstream of the murine stem cell virus promoter. DNA transfection and viral vector preparation were performed as described elsewhere [7]. HEK293T cells were transfected with transfer, envelope, and packaging plasmids by the calcium phosphate precipitation method. Viral vector particles were pelleted by centrifugation at 6,000 g for 16–18 h and resuspended in 0.1 M phosphate-buffered saline, pH 7.4 (PBS). The particles were then applied to a Sepharose Q FF ion-exchange column (GE Healthcare, Buckinghamshire, UK) in PBS and eluted with a linear 0.0–1.5 M NaCl gradient. The fractions were monitored at absorbance 260/280 nm. The peak fractions containing the particles were collected and concentrated by centrifugation through a Vivaspin filter (Vivascience, Lincoln, UK). For determination of the RNA titer, viral RNA in vector preparations was isolated with a NucleoSpin RNA virus kit (Clontech, Mountain View, CA), and the copy number of the RNA genome was determined by using a Lenti-X qRT-PCR titration kit (Clontech). PCR amplification was performed on duplicate samples by using a StepOne real-time PCR system (Applied Biosystems, Tokyo, Japan) under the following conditions: one cycle of 95°C for 3 min; and 40 cycles of 95°C for 15 s and 54°C for 1 min.

### Experimental Animals

Three Japanese monkeys (*Macaca fuscata*; monkeys M, G, and U) of either sex weighing 5.5–7.6 kg were used. The experimental protocols were approved by the Institutional Animal Care and Use Committees of Primate Research Institute, Kyoto University and National Institutes of Natural Sciences (Permission Numbers: 2011-013 and 11A125, respectively), and all experiments were conducted according to the guidelines of the National Institutes of Health Guide for the Care and Use of Laboratory Animals. All efforts were made to minimize suffering in accordance with the recommendations of the “Guidelines for Care and Use of Nonhuman Primates” (Primate Research Institute, Kyoto University). The monkeys were kept in individual primate cages in an air-conditioned room where food and water were always available. Their health conditions, including factors such as body weight and appetite, were checked daily. Supplementary fruits were provided daily.

### Surgical Procedures

Prior to the experiments, each monkey was trained to sit quietly in a monkey chair. Under general anesthesia with sodium pentobarbital (25 mg/kg, i.v.) after sedation with ketamine hydrochloride (10 mg/kg, i.m.) and xylazine hydrochloride (1–2 mg/kg, i.m.), the monkeys underwent a surgical operation to fix their head painlessly in a stereotaxic frame attached to the primate chair (for details, see [16]). Magnetic resonance imaging (3T; Allegra, Siemens, Erlangen, Germany) scans were performed to estimate stereotaxic coordinates of the target brain structures (i.e., the GPi and STN).

**Figure 1:**
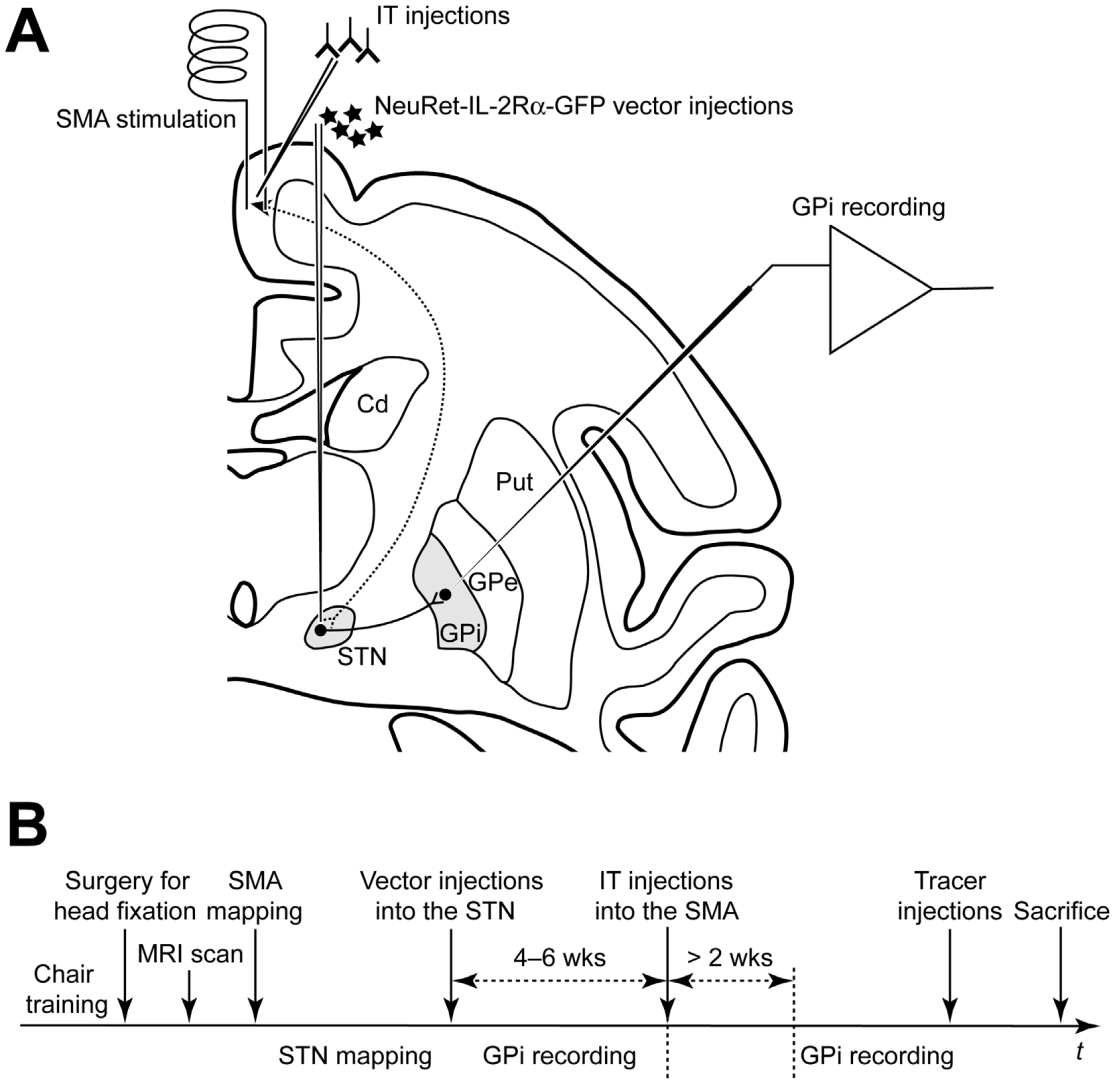
Schematic representation of the experimental setup and time-course. A: Experimental setup. A pair of bipolar stimulating electrodes was placed in the forelimb region of the supplementary motor area (SMA). A glass-coated Elgiloy-alloy microelectrode was inserted obliquely into the internal segment of the globus pallidus (GPi) for extracellular single-unit recording. The NeuRet-IL-2Rα-GFP vector was injected into the SMA-recipient territory of the subthalamic nucleus (STN) according to the mapping of cortically evoked responses therein. Four-to-six weeks later, immunotoxin (IT) was injected into the SMA forelimb region. Cd, caudate nucleus; GPe, external segment of the globus pallidus; Put, putamen. B: Experimental time-course. Neuronal recordings from the GPi in a control condition were performed before the IT injections into the SMA. Later than two weeks (wks) after the IT injections, GPi recordings were resumed.

After full recovery from the operation, the skull over the SMA was removed under light anesthesia with ketamine hydrochloride (10 mg/kg, i.m.) and xylazine hydrochloride (1–2 mg/kg, i.m.). The forelimb region and the adjacent orofacial and hindlimb regions of the SMA were identified by means of electrophysiological mapping (for details, see [9], [16]). A pair of bipolar stimulating electrodes (made of 200-µm-diameter enamel-coated stainless steel wires; intertip distance, 2 mm) was inserted into the SMA forelimb region at the depth of 4 mm (Fig. 1A). In monkey G, the electrodes were fixed chronically with transparent acrylic resin as avoiding insertion points for later IT injections into the cortex. In monkeys M and U, the stimulating electrodes were inserted in each experimental session. Finally, a rectangular plastic chamber covering the SMA was fixed onto the skull with acrylic resin.

### GPi Neuron Recordings

During the recording sessions, each monkey was in an awake state and seated in the monkey chair with his/her head restrained. A glass-coated Elgiloy-alloy microelectrode (0.5–1.5 MΩ at 1 kHz) was inserted obliquely (45° from the vertical line in the frontal plane) into the GPi using a hydraulic microdrive (Fig. 1A; for details, see [16], [17]). In the GPi, the mediodorsal aspect of its caudal part that receives input from the SMA was mainly explored [18]. Single neuron activity recorded extracellularly from the microelectrode was amplified (x 8,000), filtered (100–2,000 Hz) and displayed on an oscilloscope. The recorded activity was isolated and converted into digital pulses using a time-amplitude window discriminator. Responses of GPi neurons to bipolar electrical stimulation in the SMA (300-µs-duration single monophasic pulse, strength of less than 0.7 mA, at 0.7 Hz) were analyzed by constructing peri-stimulus time histograms (PSTHs; bin width of 1 ms; summed for 100 stimulus trials) using a computer. A typical response of GPi neurons to the SMA stimulation was a triphasic pattern composed of an early excitation, an inhibition, and a late excitation [16], [17]. Then, the spontaneous firing of GPi neurons was recorded as digitized spikes (bin width of 0.5 ms) for 50 s. Neuronal recordings from the GPi were initially performed before IT injections into the SMA as a control (Fig. 1B). Then, GPi recordings were resumed later than two weeks after the IT injections.

### STN Mapping and Vector Injections into the STN

A setup similar to that for GPi neuron recordings was used for STN mapping. A glass-coated Elgiloy-alloy microelectrode (1.0–1.5 MΩ) was inserted vertically into the STN (Fig. 1A). Neuronal responses to the SMA stimulation were analyzed by construction of PSTHs. A typical response of STN neurons was a biphasic pattern composed of an early excitation and a late excitation [16]. By mapping cortically evoked orthodromic responses, the SMA-recipient territory was identified in the STN, mainly within its medial aspect (see [9]).

Based on the results of the STN mapping, a tungusten wire electrode attached to the injection needle of a 10-µl Hamilton microsyringe was inserted vertically into the medial aspect of the STN (Fig. 1A). The tip of the needle was confirmed to be located right in the STN territory that receives input from the SMA, especially its forelimb representation, by detecting responses cortically induced through the electrode. Then, the NeuRet vector expressing IL-2Rα-GFP (NeuRet-IL-2Rα-GFP vector; 3.6×10^10^ copies/ml) was injected at 2–3 mediolaterally distinct sites (1.5 µl each) as covering the SMA-recipient territory of the STN (Fig. 1A,B).

### IT Injections into the SMA

Four-to-six weeks after the vector injections when a population of SMA neurons projecting to the STN was expected to express IL-2Rα, IT was injected into the SMA forelimb region to ablate these neurons selectively (Fig. 1A,B). For IT injection, anti-Tac(Fv)-PE38 was diluted to a final concentration of 30–40 µg/ml in PBS [19]. The injection needle of a 10-µl Hamilton microsyringe was inserted perpendicularly to the cortical surface at the depth of 3–8 mm, and the IT injections were made at multiple sites (monkeys M and U: 1.0 µl/site, two dorsoventrally separate sites per track, two tracks; monkey G: 0.5 µl/site, two dorsoventrally separate sites per track, three tracks).

### Tracer Injections

For retrograde neuronal labeling (monkey M), Fluoro-ruby (FR; Invitorogen, Eugene, OR; 10% aqueous solution) was injected into the STN while recordings of neuronal responses to the SMA stimulation. A single injection of 1.0 µl of FR was made to cover the SMA-recipient territory of the STN. For anterograde tract tracing (monkeys G and U), multiple injections of biotinylated dextran amine (BDA; Invitorogen; 3,000 MW; 20% aqueous solution) were performed into the forelimb region of the SMA (0.75 µl/site, two dorsoventrally separate sites per track, three tracks) through a 10-µl Hamilton microsyringe.

### Histological Analysis

Four weeks after the tracer injections, the monkeys were deeply anesthetized with an overdose of sodium pentobarbital (50 mg/kg, i.v.) and transcardially perfused with PBS, followed by 10% formalin in 0.1 M phosphate buffer, pH 7.4 (PB). The brains were removed from the skull, postfixed in the same fresh fixative overnight, saturated with 30% sucrose in PB at 4°C, and then cut serially into 50-µm-thick frontal sections on a freezing microtome. For visualization of immunoreactive signals of FR and NeuN, a series of every eighth section was immersed in 1% skim milk for 1 h at room temperature and incubated overnight at 4°C with rabbit anti-tetramethylrhodamine antibody (Invitrogen; diluted at 1∶2,000) or mouse monoclonal anti-NeuN antibody (Millipore, Temecula, CA; diluted at 1∶4,000) in PBS containing 0.1% Triton X-100 and 1% normal goat serum. The sections were then incubated in the same fresh medium containing biotinylated goat anti-rabbit/anti-mouse IgG antibody (Vector Laboratories, Burlingame, CA; diluted at 1∶200) for 2 h at room temperature, followed by avidin-biotin-peroxidase complex (ABC Elite, Vector Laboratories) for 2 h at room temperature. Subsequently, the sections were reacted for 15±5 min in 0.05 M Tris-HCl buffer (pH 7.6) containing 0.04% diaminobenzidine (DAB), 0.04% NiCl_2_, and 0.003% H_2_O_2_. For visualization of injected and transported BDA, a series of sections was incubated in 0.05 M PBS containing 0.5% Triton X-100 overnight and processed for the ABC method as described above. Finally, the sections were mounted onto gelatin-coated glass slides, air-dried, counterstained with 1% Neutral red, and coverslipped. Another series of sections was Nissl-stained with 1% Cresyl violet.

Images of sections were digitally captured using an optical microscope equipped with a high-grade charge-coupled device (CCD) camera (Biorevo, Keyence, Osaka, Japan). The number of FR-positive neurons in the SMA was counted with a computer-assisted imaging program (BZ-II, Keyence). Seventeen sections for the SMA obtained from Monkey M were used for cell counts, and the average number per section was calculated.

### Data Analysis

**Figure 2:**
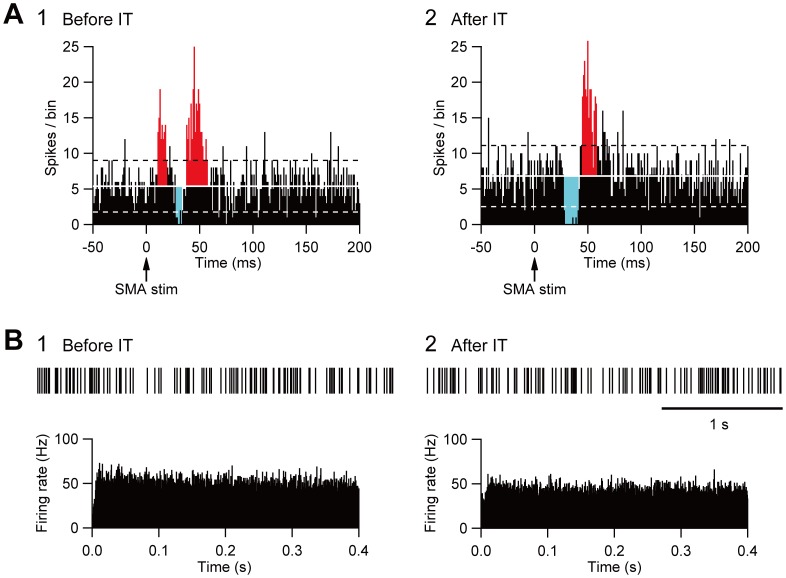
Effects on GPi neuron responses of selective elimination of the cortico-STN projection. A: Typical examples of peri-stimulus time histograms (PSTHs; bin width of 1 ms, summed for 100 stimulus trials) in monkey G before (1) and after (2) IT injections into the SMA. Electrical stimulation in the SMA was given at time  = 0 (arrows). In each PSTH, the mean firing rate and statistical levels of P<0.05 (one-tailed *t*-test) calculated from the firing rate during 100 ms preceding the onset of SMA stimulation are indicated by the solid white line (mean), broken black line (upper limit of P<0.05), and broken white line (lower limit of P<0.05), respectively. The excitatory and inhibitory responses are represented in red or cyan color, respectively. The amplitude of each response was defined as the number of spikes in the colored area. B: Typical examples of spontaneous activities denoted by slow traces of digitized spikes and autocorrelograms (bin width of 0.5 ms) in monkey G before (1) and after (2) IT injections into the SMA.

The responses of GPi neurons to the SMA stimulation were assessed with PSTHs. The mean value and standard deviation (SD) of the firing rate during 100 ms preceding the onset of stimulation were calculated from a PSTH and were considered a value for a baseline discharge. Changes in the firing rate in response to the SMA stimulation (i.e., excitation and inhibition) were judged to be significant if the firing rate during at least two consecutive bins (2 ms) reached the statistical level of P<0.05 (one tailed *t*-test), compared with the baseline discharge. The latency of each response was defined as the time for the first bin exceeding this level. Responses were judged to end when two consecutive bins fell below the significant level. The end point was determined as the time for the last bin exceeding this level. The amplitude of each response was defined as the number of spikes during the significant change minus that of the baseline discharge (i.e., the area of the response; see the colored area in Fig. 2A). For population PSTHs, the PSTH of each neuron was smoothed with a Gaussian window (σ = 2 ms) and averaged separately for monkeys and conditions (before and after the IT injections). The spontaneous firing rate and several measures of the firing pattern were calculated from 50 s of digitized recordings [20]. For firing pattern analyses, the following parameters were used: interspike intervals (ISIs); coefficient of variation (CV), kurtosis and skewness of ISIs; burst index (a ratio of the mean ISI to the mode ISI) [21]; the percentage of spikes in bursts detected by the Poisson surprise method (Poisson surprise value >3.0 with base 10; the minimum number of spikes during bursts was three) [20], [22]. In addition, the spontaneous firing pattern was analyzed by constructing autocorrelograms (bin width of 0.5 ms) from 50 s of digitized recordings.

### Safety Issues

All experimental procedures were performed in a special laboratory (biosafety level 2) designed for *in vivo* virus experiments. To avoid accidental infection, all investigators wore protective clothes during experimental sessions in the laboratory. Equipment was disinfected with 70% ethanol after each experimental session, and waste was autoclaved before disposal.

## Results

The NeuRet-IL-2Rα-GFP vector was injected into the STN of monkeys M, G, and U based on the results of the electrophysiological mapping. Histological examination showed that the vector injections were successfully made into the STN of monkeys M and G. In these monkeys, the sites of multiple injections were located in the medial aspect of the STN that receives major input from the SMA (Fig. S1A; see [9]). On the other hand, as the vector injections missed the STN in monkey U, this monkey was used as a control case.

### Cortically Evoked Responses of GPi Neurons

Following the vector injections into the STN, activity of 35 GPi neurons was recorded in monkeys M (n = 15) and G (n = 20). Among them, electrical stimulation in the SMA induced responses in 13 GPi neurons (monkey M, n = 4; monkey G, n = 9). The most popular response pattern was a triphasic response consisting of an early excitation, a subsequent inhibition, and a late excitation (12/13; Fig. 2A1) as previously reported in normal monkeys [16], [17]. This indicated that the vector injections into the STN did not affect cortically evoked responses of GPi neurons. Using immnostaining for the neuronal marker NeuN, we also confirmed that there was no conspicuous damage to the STN (Fig. S1B).

**Figure 3:**
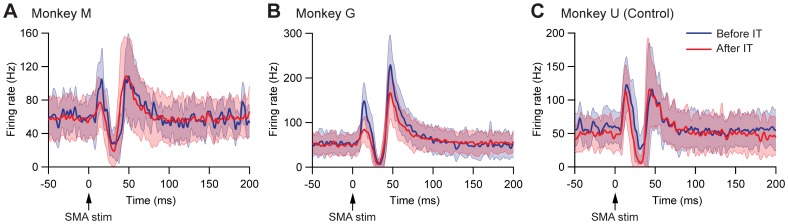
Population histograms of GPi neuron responses. Data obtained before (blue lines) and after (red lines) IT injections into the SMA in monkey M (A; before IT, n = 4; after IT, n = 18), monkey G (B; before IT, n = 9; after IT, n = 13), and monkey U (C; before IT, n = 9; after IT, n = 10). The light shaded colors represent ±1 SD. A,B: The early excitation was diminished without either the inhibition or the late excitation affected. C: Control case without vector injections into the STN. No apparent changes were observed after the IT injections into the SMA.

After IT injections into the SMA, especially its forelimb region, activity of 72 GPi neurons was recorded in monkeys M (n = 44) and G (n = 28). Among them, the SMA stimulation induced responses in 31 GPi neurons (monkey M, n = 18; monkey G, n = 13). The most popular response (16/31) was a biphasic pattern, an inhibition followed by a late excitation without an early excitation (Fig. 2A2). Other response patterns, such as a triphasic pattern of an early excitation, an inhibition, and a late excitation (9/31) and a monophasic inhibition (4/31), were also observed. The amplitude of each component was compared before and after the IT injections (Table 1). Compared with a control condition (before the IT injections), the amplitude of the early excitation was reduced to 12% of the control after the IT injections (*t*-test, P<0.01). On the other hand, the amplitude of the inhibition and the late excitation remained unchanged, although the late excitation in monkey G slightly decreased with no significant change. In addition, virtually no alterations were found in the latency of the inhibition (mean ± SD; before IT, 28.4±2.2 ms; after IT, 27.3±3.1 ms) or the late excitation (before IT, 43.2±3.1 ms; after IT, 43.7±4.9 ms), or the duration of the inhibition (before IT, 8.5±3.6 ms; after IT, 8.0±4.7 ms) or the late excitation (before IT, 17.0±8.2 ms; after IT, 15.9±8.5 ms). The amplitude change in the early excitation was further detected in population histograms (Fig. 3A,B). In monkeys M and G, the amplitude of the early excitation was greatly reduced, while the amplitude of the inhibition and the late excitation remained relatively unchanged. In monkey U in which no vector injections were made into the STN, activity of GPi neurons was recorded before (n = 17) and after (n = 19) the IT injections. Among them, nine neurons before the IT injections and 10 neurons after the IT injections responded to the SMA stimulation. The most popular response was a triphasic pattern of an early excitation, an inhibition, and a late excitation both before (7/9) and after (9/10) the IT injections into the SMA. The amplitude of each component remained intact after the IT injections (Fig. 3C, Table 1). Thus, the IT injections into the SMA combined with the NeuRet-IL-2Rα-GFP vector injections into the STN abolished the cortically evoked early excitation in the GPi without affecting either the inhibition or the late excitation.

**Table 1:** Amplitude of cortically-induced triphasic responses (early excitation, inhibition, and late excitation) of GPi neurons before and after IT injections into the SMA.

		Before IT	After IT
Early excitation	Monkey M	224±123 (n = 4)	24±47* (n = 18)
	Monkey G	788±185 (n = 9)	142±178* (n = 13)
	Monkey U^C^	435±222 (n = 9)	368±223 (n = 10)
Inhibition	Monkey M	−336±280	−379±280
	Monkey G	−414±227	−419±311
	Monkey U^C^	−423±352	−510±239
Late excitation	Monkey M	493±170	402±635
	Monkey G	2228±903	1580±1148
	Monkey U^C^	1144±845	1027±879

Each value represents the mean ± SD expressed in the number of spikes during the significant change minus that of the baseline discharge (see Fig. 2A).

* Significant decrease in comparison with data obtained before IT injections (P<0.01, *t*-test).

C Used as control.

### Spontaneous Activity of GPi Neurons

Furthermore, the spontaneous firing rate and pattern were compared before and after the IT injections into the SMA. The spontaneous firing rate (before IT, 45.8±7.1 Hz, n = 13; after IT, 46.6±12.9 Hz, n = 31) was left intact (Table 2). Other parameters, such as ISIs, CV, kurtosis and skewness of ISIs, burst index, and the percentage of spikes in bursts, did not show any significant changes (Table 2). Neurons in the GPi fired randomly at high frequency before the IT injections (Fig. 2B1), and no apparent changes were observed after the IT injections (Fig. 2B2). These results suggested that the firing rate and pattern of GPi neurons remained unchanged even after the removal of the cortico-STN projection.

**Table 2:** Firing patterns of GPi neurons before and after IT injections into the SMA.

		Before IT	After IT
Firing rate (Hz)	Monkey M	52.9±4.7 (n = 4)	49.4±14.0 (n = 18)
	Monkey G	41.7±4.4 (n = 9)	39.9±6.3 (n = 13)
	Monkey U^C^	47.2±8.8 (n = 9)	42.8±10.7 (n = 10)
ISI (ms)	Monkey M	15.9±1.2	21.7±5.7
	Monkey G	24.2±2.5	25.6±3.8
	Monkey U^C^	21.9±4.2	24.7±5.9
CV of ISI	Monkey M	1.1±0.2	1.3±0.5
	Monkey G	1.5±0.9	1.3±0.2
	Monkey U^C^	1.1±0.3	1.5±0.8
Kurtosis of ISI	Monkey M	36.1±47.8	70.3±89.1
	Monkey G	61.3±55.8	36.7±18.5
	Monkey U^C^	40.3±50.0	66.1±63.6
Skewness of ISI	Monkey M	4.2±2.9	5.6±3.6
	Monkey G	5.8±3.5	4.4±1.1
	Monkey U^C^	4.0±2.7	6.7±5.1
Busrt index	Monkey M	5.2±3.1	3.9±2.4
	Monkey G	2.7±0.8	3.1±1.6
	Monkey U^C^	4.8±2.7	3.1±2.7
Percentage of spikes in bursts (%)	Monkey M	3.2±1.0	4.7±2.7
	Monkey G	4.4±4.1	6.2±4.7
	Monkey U^C^	2.5±1.6	3.9±2.3

Each value represents the mean ± SD.

No significant changes were detected in comparison with data obtained before IT injections (P<0.01, *t*-test).

C Used as control.

### Histological Analysis

**Figure 4:**
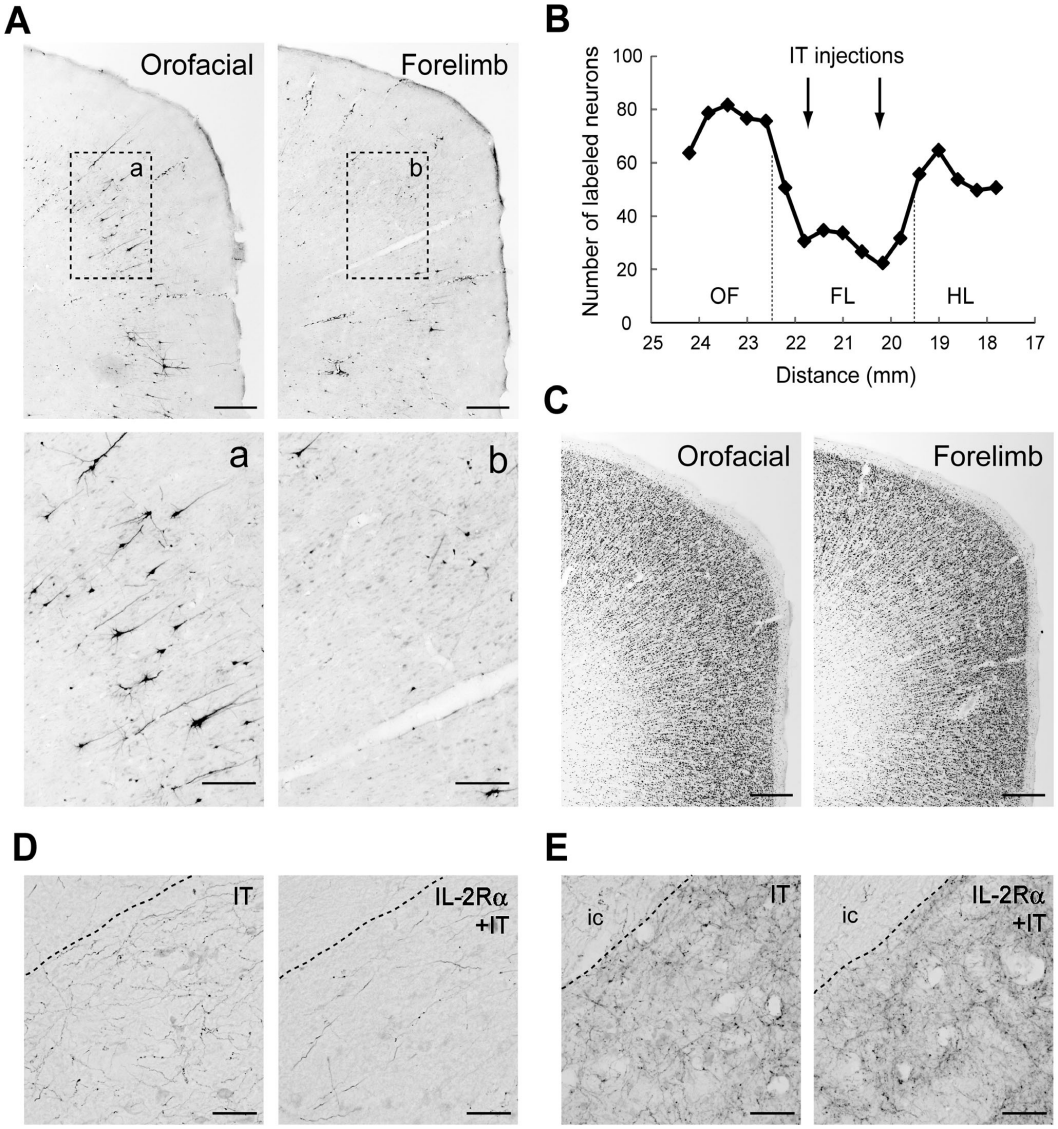
Retrograde and anterograde neural tracings. A: Retrograde neuronal labeling in the orofacial and forelimb regions of the SMA after Fluoro-ruby (FR) injection into the STN in monkey M. Lower panels a,b denote higher-power magnifications of the rectangular areas in upper panels. Scale bars, 500 µm for upper panels and 200 µm for lower panels. B: Distribution of FR-positive neurons in the orofacial (OF), forelimb (FL), and hindlimb (HL) regions of the SMA. Cell counts were carried out in 17 equidistant frontal sections taken from Monkey M. Note that FR-positive neurons are so few in the FL region where IT injections were made (at two rostrocaudally distinct levels pointed to by arrows), as compared to those in the OF and HL regions. Numerals on the abscissa represent the distance from rostrocaudal zero on the stereotaxic frame (equivalent to the interaural line). C: NeuN immunostaining of the orofacial and forelimb regions of the SMA. Note that the IT injections cause no marked neurotoxic insult. Scale bars, 500 µm. D: Anterograde terminal labeling in the STN after BDA injections into the SMA forelimb region. Left: monkey U without vector injections into the STN. Right: monkey G with vector injections into the STN. Note that BDA-labeled axon terminals are much less densely observed in the STN after the IT injections into the SMA combined with the vector (IL-2Rα) injections into the STN. Scale bars, 50 µm. E: Terminal labeling in the putamen in monkeys U (left) and G (right). Note that virtually no difference in the density of labeled terminals is found between the two cases. ic, internal capsule. Scale bars, 50 µm.

In monkeys M and G subjected to the disappearance of the early excitation responding to the SMA stimulation, retrograde and anterograde neural tracings were performed through injecting FR into the STN and BDA into the SMA. After the FR injection into the STN (monkey M), retrogradely labeled neurons in the SMA were much fewer in the forelimb region where the IT injections were primarily directed than in the orofacial and hindlimb regions (Fig. 4A,B). Moreover, immnostaining for NeuN revealed that the IT injections into the SMA caused no marked neurotoxic insult (Fig. 4C). After the BDA injections into the SMA forelimb region (monkey G), anterogradely labeled axon terminals were so largely decreased in the STN (Fig. 4D, right), as compared to the control case, monkey U (Fig. 4D, left). In remarkable contrast, dense terminal labeling from the SMA was seen in the striatum, especially the putamen, as in the control case (Fig. 4E). These anatomical data clearly indicated that cortico-STN projection originating from the SMA forelimb region was selectively eliminated without affecting either the corticostriatal projection or the cortico-STN projections from SMA regions with other representations than the forelimb.

Since the present study was primarily designed to develop a new methodology for selective removal of a given pathway, no behavioral alterations induced by the elimination of the cortico-STN projection were closely investigated. However, no apparent motor deficits were observed in either monkey M or monkey G.

## Discussion

Taking advantage of the NeuRet vector that permits highly-efficient retrograde gene-transfer with improved neuronal specificity [7], we established IT-mediated tract targeting in the primate brain. In the present study, we applied this pathway-selective targeting technique to the hyperdirect pathway. Together with the direct and indirect pathways (for reviews, see [23], [24]), the hyperdirect pathway is known to be among the major pathways of the basal ganglia [9], [10]. This pathway connects the motor-related areas of the frontal lobe to the GPi, an output station of the basal ganglia, at short latency via the STN without relay at the striatum and is involved in motor information processing in the basal ganglia. When single neuron activity was recorded in the monkey GPi in response to electrical stimulation in the motor-related areas, such as the MI and SMA, triphasic responses consisting of an early (short-latency) excitation, an inhibition, and a late (long-latency) excitation were obtained. It has been suggested that the early excitation may be derived from the cortico-STN-GPi hyperdirect pathway based on the following pharmacophysiological data (see also [10]): (1) Blockade of STN neuron activity by injection of the GABA_A_ receptor agonist, muscimol, thereinto abolished the early as well as the late excitation of GPi neurons [16]; (2) Blockade of glutamatergic input from the STN to the GPi by local injection of an ionotropic glutamatergic receptor antagonist diminished the early as well as the late excitation of GPi neurons [17]. However, these approaches concurrently decreased the firing rate of GPi neurons and changed their firing pattern from a random to a bursting and/or oscillatory one.

For selective removal of the hyperdirect pathway, the NeuRet-IL-2Rα-GFP vector was injected into the STN, and, subsequently, IT was injected into the SMA in the present experiments. Our histological examination clearly indicated that cortical neurons in the forelimb region of the SMA projecting to the STN were selectively ablated. In such model monkeys, neuronal activity in the GPi was recorded in response to electrical stimulation in the SMA. The SMA stimulation results in selective activation of SMA-recipient zones in the basal ganglia [25]. We found that out of the triphasic responses, only the early excitation was largely suppressed with the inhibition or the late excitation virtually unaffected. This indicates that IT-mediated tract targeting successfully eliminated the hyperdirect pathway selectively from the basal ganglia circuitry. The present results define that the cortically-driven early excitation of GPi neurons is derived from the cortico-STN projection. It has also been revealed in the present study that the firing rate and pattern of GPi neurons remain unchanged even after the removal of the cortico-STN projection. This implies that the cortico-STN projection conveys phasic activity changes from the SMA to the GPi, but does not contribute to maintenance of tonic activity of GPi neurons. In contrast to the early excitation, the inhibition in the GPi was not affected by the elimination of the cortico-STN projection, as it can be considered that the inhibition is mediated by the cortico-striato-GPi direct pathway [17]. On the other hand, the late excitation in the GPi was slightly diminished though not significant. This late excitation is ascribable to the late excitation in the STN and is probably mediated by the cortico-striato-external pallidal segment (GPe)-STN-GPi indirect pathway. However, it has also been suggested that the late excitation in the STN is part of the prolonged excitation induced by the cortico-STN projection, which may explain a slight decrease in the late excitation in the GPi after the elimination of the cortico-STN projection [17].

According to the cortically-induced triphasic response pattern detected in GPi neurons, the hyperdirect pathway conveys excitatory signals from the frontal motor-related areas toward the GPi, bypassing the striatum, with shorter conduction time than signals through the striatum that arise from both the direct and the indirect pathways (see [10], [16]). In favor of a dynamic “center-surround model” of basal ganglia function that was first proposed by Mink and Thach [12], we have implicated the functional role of the hyperdirect pathway in the control of voluntary limb movements (see also [10], [14], [26]). When a voluntary movement is about to be initiated by the motor cortical mechanism, a corollary signal conveyed through the cortico-STN-GPi hyperdirect pathway first inhibits large areas of the thalamic and cortical target structures that are involved not only in a desired motor program, but also in other competing programs. Then, another corollary signal through the cortico-striato-GPi direct pathway disinhibits part of the thalamic and cortical target areas and releases the desired motor program alone. Finally, the third corollary signal transmitted by way of the cortico-striato-GPe-STN-GPi indirect pathway again inhibits the thalamic and cortical target areas extensively. By means of such sequential motor information processing, only the desired motor program is initiated, executed, and terminated at appropriate timings, whereas other competing programs are canceled. Thus, it is most likely that the hyperdirect pathway exerts a powerful excitatory effect on the GPi to suppress involuntary and unnecessary movements prior to the selected motor action. The following works favor this notion: (1) Lesions or blockade of STN neuron activity induced involuntary movements, hemiballism [16], [27], [28], suggesting that both the hyperdirect and indirect pathways might inhibit unnecessary movements; (2) According to functional magnetic resonance imaging studies using human subjects, the cortico-STN projection conveys stop signals to inhibit motor responses [29], [30]; (3) It is also suggested that the cortico-STN projection may inhibit automatic movements and switch to volitionally controlled movement [31]. In the present experiments, however, no apparent behavioral changes were observed in our model monkeys subject to the IT-mediated selective removal of the hyperdirect pathway. Some specific motor task to be trained in these model monkeys may be required to disclose motor impairments.

The IT-mediated tract targeting achieves selective elimination of given pathways in monkeys as well as in rodents. This novel technique will provide a general, powerful strategy to investigate precisely not only the specific functional roles of individual pathways constituting a particular neural network, but also the large-scale operative mechanisms underlying the entire network.

## Supporting Information

Figure S1 **Frontal sections showing injection site of the NeuRet-IL-2Rα-GFP vector in the STN.** A: GFP immunostaining. B: NeuN immunostaining. Note that the vector injections were placed in the medial aspect of the STN where major input from the SMA terminates, and that there is no conspicuous damage to the STN. cp, cerebral peduncle; ot, optic tract; ZI, zona incerta. Right side, medial; upper side, dorsal. Scale bar, 1 mm.
(TIF)
