# Supplementary figures and images for: Immunotoxin-Mediated Tract Targeting in the Primate Brain: Selective Elimination of the Cortico-Subthalamic “Hyperdirect” Pathway
Source: PLoS One. 2012 Jun 25;7(6):e39149. doi: 10.1371/journal.pone.0039149 (PMC3382612; doi:10.1371/journal.pone.0039149)

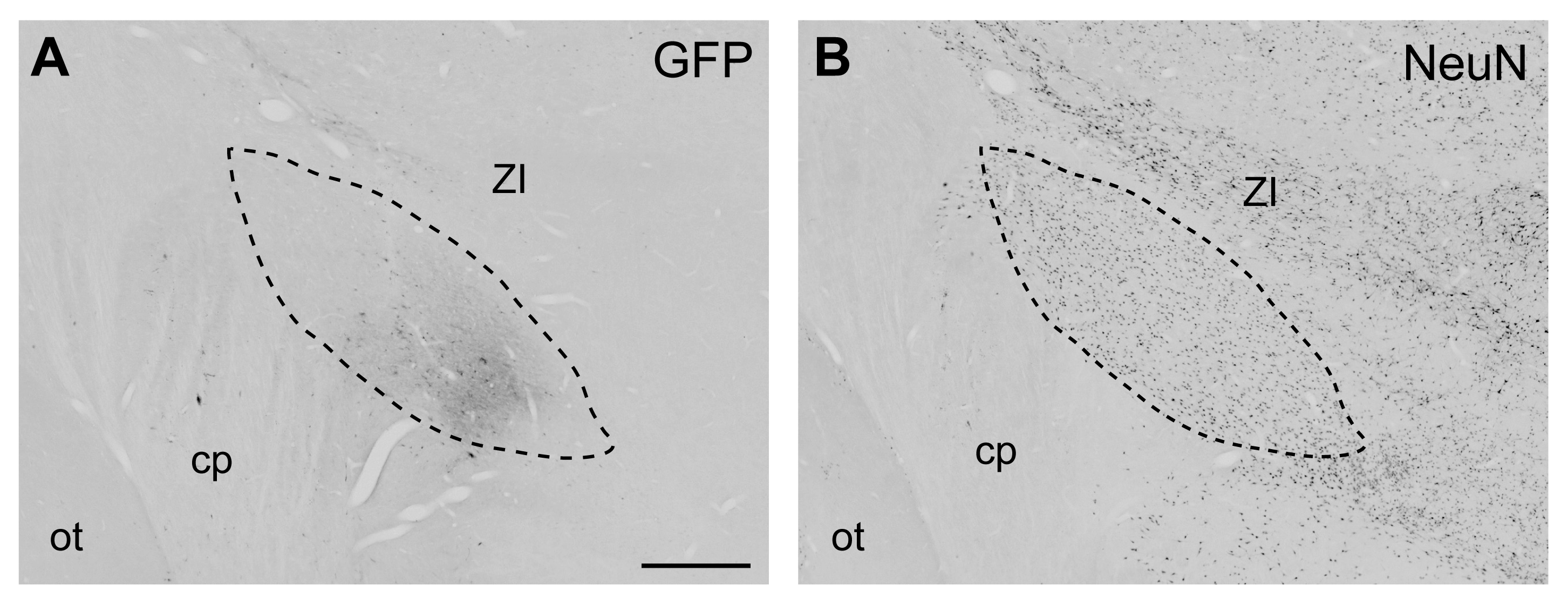

Supplement: Figure S1 — Frontal sections showing injection site of the NeuRet-IL-2Rα-GFP vector in the STN. A: GFP immunostaining. B: NeuN immunostaining. Note that the vector injections were placed in the medial aspect of the STN where major input from the SMA terminates, and that there is no conspicuous damage to the STN. cp, cerebral peduncle; ot, optic tract; ZI, zona incerta. Right side, medial; upper side, dorsal. Scale bar, 1 mm. (TIF) [file pone.0039149.s001.tif]
